# Impact of ischemic preconditioning on functional sympatholysis during handgrip exercise in humans

**DOI:** 10.14814/phy2.12304

**Published:** 2015-02-23

**Authors:** Masahiro Horiuchi, Junko Endo, Dick H J Thijssen

**Affiliations:** 1 Division of Human Environmental Science, Mt. Fuji Research InstituteFuji-yoshida city, Yamanashi, Japan; 2 Research Institute of Sport and Exercise Sciences, Liverpool John Moores UniversityLiverpool, UK; 3 Radboud University Medical Center, Radboud Institute for Health SciencesNijmegen, Netherlands

**Keywords:** Blood flow, near-infrared spectroscopy, sympathetic vasoconstriction, tissue oxygenation

## Abstract

Repeated bouts of ischemia followed by reperfusion, known as ischemic preconditioning (IPC), is found to improve exercise performance. As redistribution of blood from the inactive areas to active skeletal muscles during exercise (i.e., functional sympatholysis) is important for exercise performance, we examined the hypothesis that IPC improves functional sympatholysis in healthy, young humans. In a randomized study, 15 healthy young men performed a 10-min resting period, dynamic handgrip exercise at 10% maximal voluntary contraction (MVC), and 25% MVC. This protocol was preceded by IPC (IPC; 4 × 5-min 220-mmHg unilateral occlusion) or a sham intervention (CON; 4 × 5-min 20-mmHg unilateral occlusion). Near-infrared spectroscopy was used to assess changes in oxygenated hemoglobin and myoglobin in skeletal muscle (HbO_2_ + MbO_2_) in response to sympathetic activation (via cold pressor test (CPT)) at baseline and during handgrip exercise (at 10% and 25%). In resting conditions, HbO_2_ + MbO_2_ significantly decreased during CPT (−11.0 ± 1.0%), which was significantly larger during the IPC-trial (−13.8 ± 1.2%, *P *= 0.006). During handgrip exercise at 10% MVC, changes in HbO_2_ + MbO_2_ in response to the CPT were blunted after IPC (−8.8 ± 1.5%) and CON (−8.3 ± 0.4%, *P *= 0.593). During handgrip exercise at 25% MVC, HbO_2_ + MbO_2_ in response to the CPT increased (2.0 ± 0.4%), whereas this response was significantly larger when preceded by IPC (4.2 ± 0.6%, *P *= 0.027). Collectively, these results indicate that IPC-induced different vascular changes at rest and during moderate exercise in response to sympathetic activation. This suggests that, in healthy volunteers, exposure to IPC may alter tissue oxygenation during sympathetic stimulation at rest and during exercise.

## Introduction

Recent studies have reported that exposure to ischemic preconditioning (IPC) can improve cycle, running, and swim exercise performance (de Groot et al. 2010; Jean-St-Michel et al. 2011; Bailey et al. 2012). IPC relates to repeated periods of ischemia followed by reperfusion which is originally described to delay cardiac cell injury (Murry et al. 1986) and protect against myocardial and vascular damage (Eisen et al. 2004). Although the working mechanisms how IPC may influence exercise performance are largely unknown, some of the beneficial impact may relate to a direct effect of IPC on the vasculature. For example, (repeated) IPC may improve vascular function and perfusion locally (Kimura et al. 2007; Jones et al. 2014), but also enhances perfusion in the contra-lateral limb (Kimura et al. 2007) and increases flow velocity of the coronary vessels (Shimizu et al. 2007; Zhou et al. 2007). Furthermore, Bailey et al. (2012) found that acute IPC could prevent the decline in vascular function that is typically observed after strenuous exercise.

Functional sympatholysis represents an important phenomenon in exercise performance and refers to the blunting of sympathetically mediated vasoconstriction during exercise. At the onset of exercise, activation of the sympathetic nervous system results in a systemic vasoconstriction. Accumulation of metabolites in exercising skeletal muscle, however, locally blunts the sympathetically driven vasoconstriction in the active muscles allowing for a marked increase in blood flow to the active muscles (Remensnyder et al. 1962). Recent studies in animals (Jendzjowsky and Delorey 2013; Mizuno et al. 2014) and humans (Mortensen et al. 2014) demonstrated that exercise training improves functional sympatholysis, additionally increases in maximal oxygen uptake (Mizuno et al. 2014). Therefore, functional sympatholysis may contribute to exercise performance.

The benefits of IPC to alter exercise performance may relate to the effects of IPC on the vasculature under resting conditions (Kimura et al. 2007; Shimizu et al. 2007; Zhou et al. 2007; Jones et al. 2014) and during exercise (de Groot et al. 2010; Jean-St-Michel et al. 2011; Bailey et al. 2012). Interestingly, animal studies have demonstrated that IPC can activate the K_ATP_ channels (Pell et al. 1998; Miura et al. 2001; Dickson et al. 2002), whereas others have suggested that functional sympatholysis is, at least partly, related to activation of K_ATP_ channels (Thomas et al. 1997; Keller et al. 2004). Therefore, the purpose of our study was to examine the potential impact of IPC (applied to the arms) on functional sympatholysis during exercise in healthy young humans. For this purpose, near-infrared spectroscopy (NIRS) was used to evaluate oxygenated hemoglobin and myoglobin (HbO_2_ + MbO_2_) changes with high temporal resolution in the forearm during handgrip exercise with and without sympathetic vasoconstriction (Hansen et al. 1996; Fadel et al. 2004b; Horiuchi et al. 2014). In this regard, changes in HbO_2_ + MbO_2_ have been shown to provide a reliable measure of sympathetic vasoconstriction in resting and steady-state exercising skeletal muscle (Fadel et al. 2004a). We hypothesized that, relative to rest, IPC will attenuate the sympathetically mediated reduction in muscle oxygenation during exercise.

## Methods

### Subjects

Fifteen healthy male subjects with a mean age of 22±3 years (means ± SD), height of 172 ± 5 cm and body weight of 67±5 kg participated in this study. Subjects did not engage in regular physical activity. After a detailed description and explanation of all study procedures and the possible risks and benefits of participation, each subject signed an informed consent form. All subjects underwent a familiarization session to become accustomed to experimental measurements and procedures. Subjects were requested to abstain from caffeinated beverages for 12 h and from strenuous exercise and alcohol for a minimum of 24 h before any experimental sessions. All procedures were approved by the ethical committee of Mt. Fuji Research Institute in Japan and were performed in accordance with the guidelines of the Declaration of Helsinki. All studies were performed at an ambient temperature of 24 ± 2°C with external stimuli minimized.

### Experimental design

Each subject visited six times to our laboratory to perform the experimental procedure. All subjects performed three different protocols; (1) 10 min resting period test, (2) dynamic handgrip exercise (40 repetitions/min) at 10% maximal voluntary contraction (MVC; light exercise), and (3) dynamic handgrip exercise (40 repetitions/min) at 25% MVC (moderate-intensity exercise), which were preceded by four cycles of 5 min unilateral cuff inflation of the upper arm to 220 mmHg (IPC) or to 20 mmHg (CON). This model of localized handgrip exercise, NIRS and sympathetic stimulation to examine functional sympatholysis has been adopted in previous studies and was demonstrated to be a reproducible method (Hansen et al. 1996).

**Figure 1:**
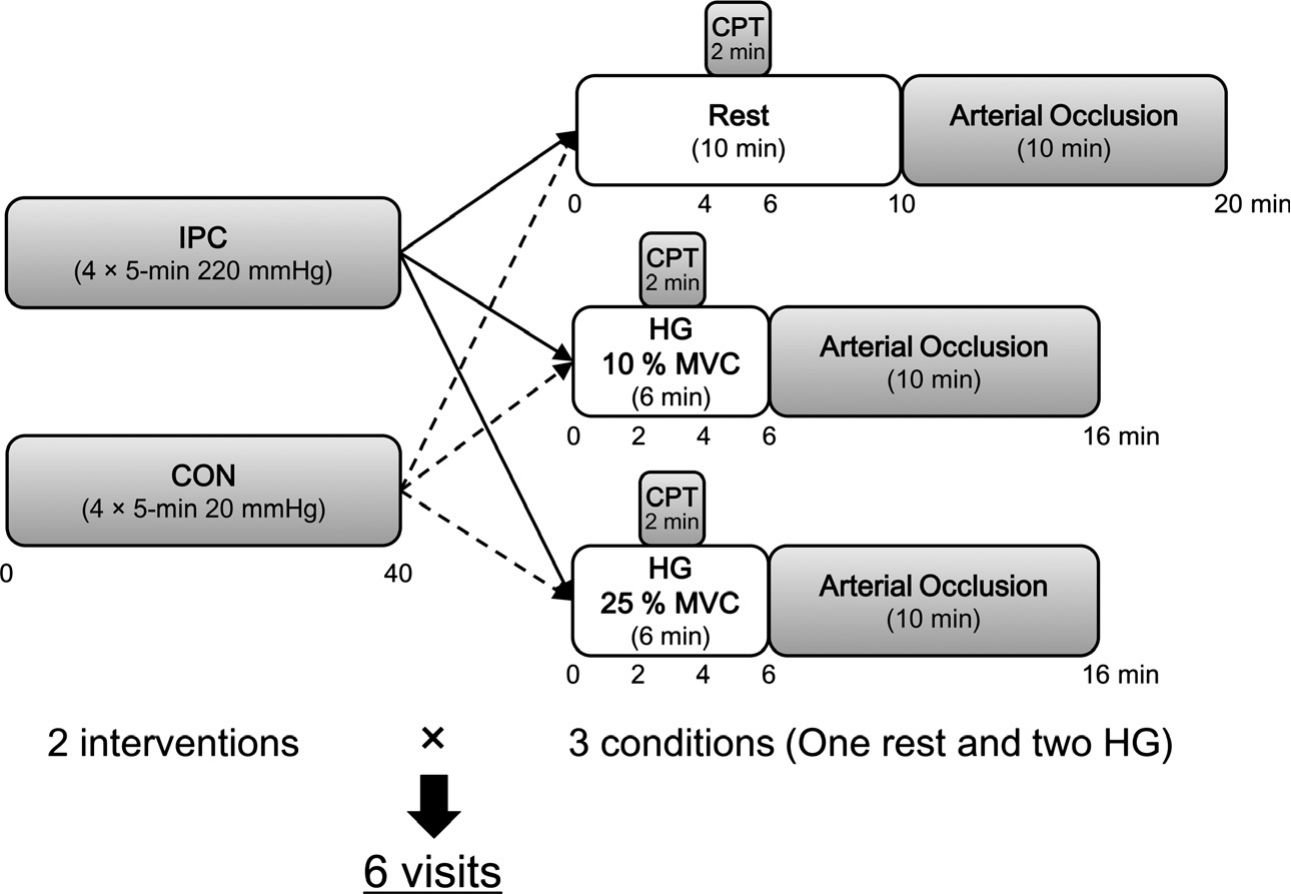
Protocol of the study. IPC, ischemic preconditioning (220 mmHg) 4 × 5-min upper arm cuff occlusion; CON, control intervention, reduced cuff pressure of 20 mmHg; MVC, maximal voluntary contraction; CPT, cold pressor test up to ankle with ice water. In total, two intervention (IPC or CON) times three condition (Rest, 10%, and 25% MVC) equals six trails were performed for each subject.

A cold pressor test (CPT) was performed during the 10-min resting period (minute 4–6) and during the 6-min dynamic handgrip exercise period (minute 2–4) to increase muscle sympathetic nerve activity (Victor et al. 1987). Several previous studies have used this protocol to evaluate sympatholysis (Parker et al. 2007; Wray et al. 2007; Horiuchi et al. 2014), because CPT is a potent, reproducible stimulus to increase muscle sympathetic outflow (Victor et al. 1987) without influencing skin sympathetic nerve activity (Fagius et al. 1989). Subjects were instructed to immerse their foot, up to the ankle, in a mixture of ice and water (3°C) for 2 min. The protocol of our study is shown in Fig.1.

All subjects performed a familiarization trial to get customized to the procedures and performing handgrip at 10% and 25% MVC. During dynamic handgrip exercise, force production was displayed on the monitor of a desktop computer to provide of subjects visual feedback. Each protocol trial was separated by at least a 48–72 h to avoid multiple effects of IPC (Loukogeorgakis et al. 2005) and was administered in a randomized order.

### Experimental measurements

#### Maximal Voluntary Contraction (MVC)

Isometric handgrip strength of the dominant arm was measured three times, with the subject in a sitting position, using a custom-made dynamometer (EKJ094; EVERNEW, Tokyo, Japan) and digital indicator (TD-510; TEAC, Tokyo, Japan). Each subject held the dynamometer and was encouraged to exert the strongest possible force for 3 sec. The intervals between each trial were set for 5 min. The MVC was defined as the mean value of the three trials.

#### Central Cardiovascular Measures

Systolic and diastolic arterial blood pressure (BP) was measured every minute at rest and during exercise using an automated blood pressure monitoring system (HEM7420; Omron, Tokyo, Japan) and heart rate (HR) was measured continuously using a three-lead electrocardiogram.

#### Skeletal Muscle Oxygenation

Local tissue oxygenation profiles of the flexor digitorum superficialis muscle were measured using NIRS (BOM-L1TRW; Omega Wave, Japan), as previously described (Horiuchi et al. 2014). This instrument uses three laser diodes (780, 810, and 830 nm), and calculates relative tissue levels of HbO_2_ + MbO_2_, and deoxygenated hemoglobin and myoglobin (Hb + Mb) according to the modified Beer–Lambert law. Also, because large vessels (>1 mm in diameter) contain sufficient hemoglobin to maximally absorb NIR light, the NIRS signals reflect changes in light absorption by hemoglobin in the small arterioles, capillaries, and venules of the microcirculation (Mancini et al. 1994). Therefore, during steady-state conditions when oxygen utilization is constant, changes in the NIR signal should primarily reflect changes in oxygen delivery or blood flow at the level of microcirculation (Mancini et al. 1994; Fadel et al. 2004a). NIRS optodes were placed on the flexor digitorum superficialis muscles, as in our previous study (Miyazawa et al. 2012). Skin and adipose tissue thickness was measured by Doppler B-mode ultrasound (logic-e, GE health care, Japan) to guide accurate placement of the NIRS optodes and measure skin blood flow (SkBF). These measures were needed to verify that the NIRS signal was assessing changes in the desired muscle bed. Briefly, the measurement depth of the NIR signal was about half the distance between the two fiber optic bundles placed over the skin, one comprising the light source and the detector (Patterson et al. 1989). With this in mind, we used a distance of 4 cm between probes, which would provide a NIRS signal traversing approximately 20 mm. This would have allowed the appropriate depth to sample from the flexor digitorum superficialis muscle, as the skinfold thickness was 4.8 ± 0.3 mm and the muscle thickness was 19.7 ± 0.7 mm. These numbers indicate that when NIR probes were placed over the skin of the muscle, the NIR light was indeed transmitted to the desired muscle bed.

The NIRS optodes were housed in a rubber holder, ensuring that the relative position of the optodes was fixed and invariant. The skin under the probes was carefully shaved and the optodes assembly was secured on the skin surface with tape and then covered with a black cloth, minimizing the intrusion of extraneous light and loss of NIR-transmitted light from the field of interrogation. The flexor digitorum superficialis muscle, with attached optodes and covering, was wrapped with an elastic bandage to minimize movement of the optodes, while still permitting freedom of movement for handgrip exercise. Pen marks were made over the skin to indicate the margins of the holder to verify that the optodes remained in place for each exercise test. No sliding was observed in any subject at the end of the exercise tests.

#### Forearm Skin Blood Flow (SkBF)

Forearm Skin Blood Flow (SkBF): SkBF was recorded from the flexor digitorum superficialis muscle using the laser-Dopper method (ATBF-LC1; Unique Medical Co. Ltd. Tokyo, Japan). Briefly, after determining the flexor digitorum superficialis muscle using Doppler ultrasound, as described in detailed above, the skin was carefully shaved and cleaned with alcohol, prior to placement of the electrodes. Signals were stored with a sampling frequency of 1 kHz on the hard disk of a personal computer.

### Data analysis

**Figure 2:**
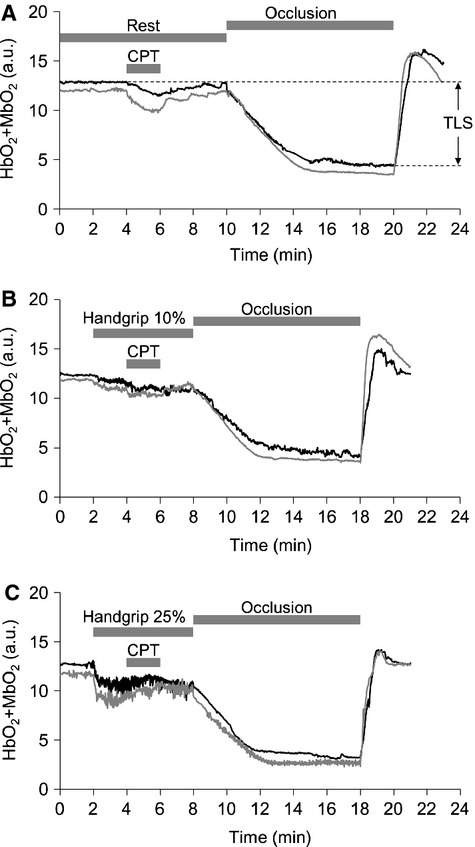
Original records showing HbO_2_ + MbO_2_ responses to CPT at rest (A) and during 10% (B), and 25% (C) MVC in one subject. Black line represents under CON and gray line represents under IPC in arbitrary unit (a.u.). TLS is total labile signal. Area under the curve represents HbO_2_ + MbO_2_ responses to acute sympathetic stimulation (CPT).

To compare NIRS signals between subjects, the changes in HbO_2_ + MbO_2_ and Hb + Mb were quantified as a percentage of the total labile signal (TLS), as previously described in detail (Hansen et al. 1996; Fadel et al. 2004a,b; Horiuchi et al. 2014). The TLS was defined in each experiment as the difference between the baseline and complete deoxygenation produced by inflation of a pneumatic cuff (MT-860; Mizuho-Medical Co. Ltd, Nagoya, Japan) on the upper arm to 300 mmHg after each protocol and maintained for 10 min (Hansen et al. 1996; Fadel et al. 2004a,b; Horiuchi et al. 2014). In this regard, HbO_2_ + MbO_2_ at baseline was defined as 100% and at physiological minimum value was defined as 0% (Fig.2). Similarly, Hb + Mb at baseline was defined as 0% and at physiological minimum value was defined as 100%. In addition, to compare SkBF between subjects, an analysis similar to NIRS signals was used, that is, resting baseline values of SkBF was defined as 100% and minimum value during arterial occlusion was defined as 0%. Thus, changes in SkBF were also represented as relative changes from baseline values.

To evaluate changes in HbO_2_ + MbO_2_ signals in response to acute sympathetic stimulation (i.e., sympatholysis) induced by the CPT, we used the differences in 20 sec steady-state averages of HbO_2_ + MbO_2_ signals just prior to the CPT and the last 20 sec of the CPT as an index of sympathetic vasoconstriction (Hansen et al. 1996; Fadel et al. 2004a,b; Horiuchi et al. 2014).

Mean arterial pressure (MAP) was calculated as [(2 × diastolic pressure) + systolic pressure] / 3. The MAP and HR responses to exercise, that is, pre-CPT, are taken from minute 1 to 2 and the CPT is taken from minute 3 to 4.

### Statistical analysis

Data are expressed as means ± standard error of the mean (SEM). Our primary outcome was HbO_2_ + MbO_2_ changes. We used two-way repeated ANOVA (Sigma Stat ver. 3.5; Hulinks, Chicago, IL) to examine whether the IPC-intervention (IPC vs. CON) and exercise intensity (rest, 10% and 25% MVC) altered the change in HbO_2_ + MbO_2_ to the CPT. A paired t-test was used for comparison the range of the difference between maximal and minimum arbitrary unit in HbO_2_ + MbO_2_ signals within each condition (rest, 10%, and 25% MVC). A paired t-test was used for comparison in SkBF response to the CPT at rest, and Hb + Mb response to the CPT at rest, and during each exercise, because baseline values were defined as 0%, whereas two-way repeated ANOVA was performed for comparison in SkBF, within each exercise test, that is, two main effects were time (pre-CPT vs. during CPT) and condition (IPC vs. CON). Similar statistical analysis was also used for comparison in MAP, and HR, within the resting period and each exercise test. A Tukey post hoc test for two-way ANOVA was employed when interactions were significant. A *P*-value of <0.05 was considered statistically significant.

## Results

We found no significant differences in the range of arbitrary unit (a.u.) of HbO_2_ + MbO_2_ between CON and IPC at rest (CON: 8.17 ± 0.14, IPC: 8.25 ± 0.21 (a.u.)), during 10% MVC (CON: 8.13 ± 0.28, IPC: 8.17 ± 0.32 (a.u.)) and 25% MVC (CON: 9.55 ± 0.41, IPC: 9.62 ± 0.44 (a.u.)) (all *P >* 0.05). This indicates that the TLS method can be adopted to compare trials. Exercise at 25%, but not at 10% MVC, induced a small, but significant increase in HR and MAP (Table1). CPT induced a significant increase in HR and MAP, which was similarly present between rest, 10% MVC and 25% MVC (Table1).

**Table 1:** Mean arterial pressure, and heart rate responses to cold pressor test (CPT) administered at rest and during steady-state exercise

						2-way ANOVA *P* values		
			Baseline	Pre-CPT	During CPT	IPC	Time	IPC × Time
Mean arterial pressure (mmHg)	Rest	CON	–	75 ± 1	88 ± 2*	0.098	<0.001	0.536
		IPC	–	76 ± 1	90 ± 1*			
	10% MVC	CON	76 ± 1	78 ± 1	91 ± 2*	0.796	<0.001	0.313
		IPC	75 ± 1	77 ± 2	91 ± 2*			
	25% MVC	CON	76 ± 1	80 ± 1†	94 ± 2*	0.447	<0.001	0.454
		IPC	75 ± 1	80 ± 1†	93 ± 2*			
Heart rate (beats/min)	Rest	CON	–	70 ± 1	75 ± 1	0.234	<0.001	0.639
		IPC	–	70 ± 1	75 ± 1			
	10% MVC	CON	70 ± 2	72 ± 2	79 ± 2*	0.204	<0.001	0.875
		IPC	72 ± 2	73 ± 2	80 ± 2*			
	25% MVC	CON	71 ± 1	75 ± 1†	81 ± 1*	0.894	<0.001	0.656
		IPC	70 ± 2	75 ± 2†	81 ± 1*			

Values are means ± standard error of the mean (SEM). CON; control intervention, IPC; ischemic preconditioning intervention.

* *P *< 0.05 between pre-CPT and during CPT.

† *P *< 0.05 between baseline and pre-CPT.

### HbO_2_ + MbO_2_ responses to exercise and CPT

**Figure 3:**
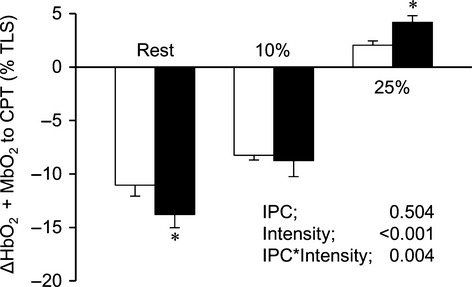
Group summary data of HbO_2_ + MbO_2_ changes in response to acute sympathetic stimulation with CPT at rest, 10% MVC, and 25% MVC. Data are means ± SEM. White bars represents CON and black bars represents IPC condition in total labile signal (TLS). *P < 0.05 between CON and IPC.

**Figure 4:**
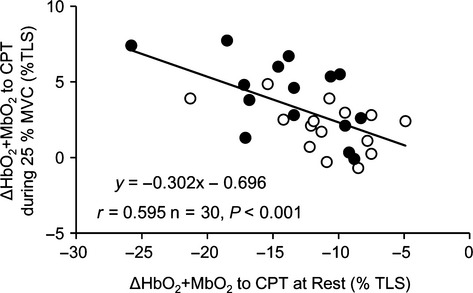
Relationship the changes in HbO_2_ + MbO_2_ in response to acute sympathetic stimulation with CPT between at rest and during moderate-intensity handgrip exercise (25% MVC). White circles represent CON and black circles represent IPC condition.

Original records showing tissue oxygenation (HbO_2_ + MbO_2_) in response to sympathetic activation evoked by CPT at rest and during 10% and 25% MVC are provided in Fig.2. During the 10% and 25% MVC trial, HbO_2_ + MbO_2_ exhibited a reduction at the onset of exercise and reached a new steady-state level within 60–90 sec of the exercise (Fig.2). At baseline, the reduction in HbO_2_ + MbO_2_ to CPT during CON (−11.0 ± 1.0%) was significantly smaller than under IPC (−13.8 ± 1.2%, *P *= 0.006). At 10% MVC handgrip exercise, CPT induced a significant decrease in HbO_2_ which was similarly present between CON (−8.3 ± 0.4%) and IPC (−8.8 ± 1.5%, *P *= 0.593). During handgrip exercise at 25% MVC, CPT caused a significant increase in HbO_2_ + MbO_2_ was present in CON (2.0 ± 0.4%), whereas this increase was significantly larger after IPC (4.2 ± 0.6%, *P *= 0.027) (Figs.2 and 3). When data were pooled, the CPT-induced changes in HbO_2_ + MbO_2_ at rest were inversely correlated with the CPT-induced changes in HbO_2_ + MbO_2_ during exercise at 25% MVC (*r* = −0.595, *P *< 0.001, *n* = 30, Fig.4).

### Hb + Mb responses to exercise and CPT

Hb + Mb increased during CPT under all three conditions. We found no significant differences in Hb + Mb responses between CON and IPC at rest (CON: 7.4 ± 1.4, IPC: 9.0 ± 1.4%), at 10% MVC (CON: 12.2 ± 1.6, IPC: 13.4 ± 1.2%), and 25% MVC (CON: 18.3 ± 0.8, IPC: 19.8 ± 1.2%) (all: *P *> 0.05).

### SkBF responses to exercise and CPT

We found no significant differences in the SkBF response to CPT at rest, during 10% MVC and 25% MVC (Interaction-effects: *P *> 0.05, Table2). Repeated measures ANOVA revealed no significant main effect of ‘time’ (pre vs. during CPT), ‘intervention’ (CON vs. IPC), or the interaction-effect of ‘time × intervention’ (Table2).

**Table 2:** Skin blood flow responses to cold pressor test (CPT) administered at rest and during steady-state exercise

						2-way ANOVA *P* values		
			Baseline	Pre-CPT	During CPT	IPC	Time	IPC × Time
Skin blood flow (%)	Rest	CON	–	100	93 ± 1	–	–	–
		IPC	–	100	94 ± 1			
	10% MVC	CON	100	117 ± 1	118 ± 1	0.256	0.189	0.599
		IPC	100	115 ± 1	117 ± 2			
	25% MVC	CON	100	128 ± 1	131 ± 3	0.822	0.067	0.522
		IPC	100	128 ± 2	133 ± 3			

Values are means ± SEM. CON, control intervention; IPC, ischemic preconditioning intervention.

## Discussion

To our knowledge, this is the first study to examine whether IPC alters functional sympatholysis in humans. In agreement with previous studies (Hansen et al. 1996; Horiuchi et al. 2014), we found that sympathetic vasoconstriction was attenuated in accordance with an increase in exercise intensity, irrespective of either IPC or CON. More importantly, we found that unilateral IPC alters the level of sympathetic vasoconstriction in response to sympathetic activation compared to CON under resting conditions (i.e., larger constriction) and during moderate-intensity exercise (i.e., larger dilation). These findings suggest that IPC alters sympathetic vasoconstriction at rest and during moderate-intensity exercise in healthy volunteers.

Under resting conditions, vasoconstrictor response to sympathetic stimulation was greater with IPC compared to CON. Previous studies have found that IPC enhances perfusion in the contra-lateral limb (Kimura et al. 2007) and increase flow velocity of (distant) coronary vessels (Shimizu et al. 2007; Zhou et al. 2007). In addition, acute exposure of remote IPC could prevent exercise-induced decrease in endothelial function (Bailey et al. 2012). When relating such findings to our observations, IPC may have caused a decrease in resting vascular tone, providing the opportunity for the CPT to evoke a greater constriction under resting conditions. Alternatively, a previous human study demonstrated that IPC can directly affect the sympathetic nervous system (Loukogeorgakis et al. 2005), that is, autonomic blockade by trimetaphan diminished a protective effect of remote IPC on endothelial function, which may explain our findings. Although the underlying mechanism remains unclear, our study suggests that IPC affects vascular responses to sympathetic activation under resting conditions.

The main purpose of our study was to assess the impact of IPC on functional sympatholysis. During light handgrip exercise, a smaller (10% MVC) or absent (25% MVC) vasoconstriction was observed during the CPT compared to resting conditions. This finding reinforces observations from previous studies (Hansen et al. 1996; Horiuchi et al. 2014) and indicates that our model was successful in identifying functional sympatholysis. Furthermore, we found that IPC did not affect the vasoconstrictor response to light intensity (i.e., 10% MVC), whereas a stronger vasodilation was observed with IPC during moderate-intensity handgrip exercise (i.e., 25% MVC). The stronger vasodilation after IPC may suggest that IPC improves redistribution of blood from inactive areas to active skeletal muscles during exercise.

These findings are interesting in light of the beneficial impact of IPC on exercise performance (de Groot et al. 2010; Jean-St-Michel et al. 2011; Bailey et al. 2012). Especially when performed at more intense, strenuous intensity, IPC seems to alter exercise performance (de Groot et al. 2010; Jean-St-Michel et al. 2011). This observation fits with our findings, in that IPC enhanced functional sympatholysis at 25% MVC, but not at the lower intensity level of 10% MVC. However, the remains speculative and should be subject for further research.

Although this study was not aimed to understand or clarify the mechanisms of IPC, there are several possible explanations for the effects observed in our study. Previous work demonstrated that activation of KATP channels may contribute to the vascular responses during functional sympatholysis (Thomas et al. 1997; Keller et al. 2004), whereas KATP channels also seem to be involved in IPC (Pell et al. 1998; Miura et al. 2001; Dickson et al. 2002; Loukogeorgakis et al. 2007). Therefore, IPC may alter functional sympatholysis through changing KATP channels. Another possible explanation relates to IPC-induced upregulation of the nitric oxide (NO) pathway (Kharbanda et al. 2001; Loukogeorgakis et al. 2005; Bailey et al. 2012). As NO antagonizes sympathetically mediated vasoconstriction through KATP channel activity (Murphy and Brayden 1995; Thomas and Victor 1998), upregulation of the NO pathway may contribute to the improved functional sympatholysis after IPC. Future studies should further examine the potential underlying mechanisms that explain the impact of IPC on functional sympatholysis.

### Methodological considerations

Although our approach is validated and frequently adopted (Parker et al. 2007; Wray et al. 2007; Horiuchi et al. 2014), some limitations must be considered. First, our NIRS data may be affected by changes in skin perfusion (Davis et al. 2006). To correct for this, we also measured SkBF. Although HbO_2_ + MbO_2_ may be affected by SkBF (Grassi et al. 2003), the observation that SkBF responses did not differ between CON and IPC indicates that our main conclusions are not importantly influenced by skin blood flow. Similarly, NIRS can represent only relative changes of HbO_2_ + MbO_2_, not blood flow. In this study, we did not measure forearm blood flow at rest and during exercise. Although this may be one of the limitations to interpret our results, NIRS has several important advantages to assess sympathetic vasoconstriction in exercising muscles. For example, the NIRS probe placement is sufficiently stable to acquire measurements even during high-intensity rhythmic contraction, and NIR signal is sufficient to reflect changes in oxygenation in the truly active small muscle groups of the forearm, that is, the flexor digitorum superficialis muscle, which can be mainly recruited during dynamic handgrip exercise (Hansen et al. 1996; Miyazawa et al. 2012). A second potential limitation is the potential impact of IPC on the range of TLS. However, our results clearly demonstrated no significant differences in Hb + Mb signals and TLS between CON and IPC. This excludes the possibility that IPC-induced changes in the Hb + Mb and HbO_2_ + MbO_2_ signals affect our main outcomes. A final limitation is that we used serial CPT tests, which may have caused desensitization (and therefore attenuation of the hemodynamic responses to CPT. To prevent such effects, all trials were counterbalanced (between IPC and CON). More importantly, CPT evoked similar increases in MAP across the various condition, which suggests that performing repeated CPTs did not affect our major outcomes.

In summary, the present results suggest, for the first time in humans, that IPC affects sympathetically mediated vasoconstriction, both at rest and during moderate-intensity dynamic handgrip exercise in healthy young men. Although speculative and future studies are warranted, these findings may contribute to the improved exercise performance that has been related to the application of IPC prior to an exercise bout.

## References

[b1] Bailey TG, Birk GK, Cable NT, Atkinson G, Green DJ, Jones H (2012). Remote ischemic preconditioning prevents reduction in brachial artery flow-mediated dilation after strenuous exercise. Am. J. Physiol. Heart Circ. Physiol.

[b2] Davis SL, Fadel PJ, Cui J, Thomas GD, Crandall CG (2006). Skin blood flow influences near-infrared spectroscopy-derived measurements of tissue oxygenation during heat stress. J. Appl. Physiol. (1985).

[b3] Dickson EW, Tubbs RJ, Porcaro WA, Lee WJ, Blehar DJ, Carraway RE (2002). Myocardial preconditioning factors evoke mesenteric ischemic tolerance via opioid receptors and K(ATP) channels. Am. J. Physiol. Heart Circ. Physiol.

[b4] Eisen A, Fisman EZ, Rubenfire M, Freimark D, McKechnie R, Tenenbaum A (2004). Ischemic preconditioning: nearly two decades of research. A comprehensive review. Atherosclerosis.

[b5] Fadel PJ, Keller DM, Watanabe H, Raven PB, Thomas GD (2004a). Noninvasive assessment of sympathetic vasoconstriction in human and rodent skeletal muscle using near-infrared spectroscopy and Doppler ultrasound. J. Appl. Physiol. (1985).

[b6] Fadel PJ, Wang Z, Watanabe H, Arbique D, Vongpatanasin W, Thomas GD (2004b). Augmented sympathetic vasoconstriction in exercising forearms of postmenopausal women is reversed by oestrogen therapy. J. Physiol.

[b7] Fagius J, Karhuvaara S, Sundlof G (1989). The cold pressor test: effects on sympathetic nerve activity in human muscle and skin nerve fascicles. Acta Physiol. Scand.

[b8] Grassi B, Pogliaghi S, Rampichini S, Quaresima V, Ferrari M, Marconi C (2003). Muscle oxygenation and pulmonary gas exchange kinetics during cycling exercise on-transitions in humans. J. Appl. Physiol. (1985).

[b9] de Groot PC, Thijssen DH, Sanchez M, Ellenkamp R, Hopman MT (2010). Ischemic preconditioning improves maximal performance in humans. Eur. J. Appl. Physiol.

[b10] Hansen J, Thomas GD, Harris SA, Parsons WJ, Victor RG (1996). Differential sympathetic neural control of oxygenation in resting and exercising human skeletal muscle. J. Clin. Invest.

[b11] Horiuchi M, Fadel PJ, Ogoh S (2014). Differential effect of sympathetic activation on tissue oxygenation in gastrocnemius and soleus muscles during exercise in humans. Exp. Physiol.

[b12] Jean-St-Michel E, Manlhiot C, Li J, Tropak M, Michelsen MM, Schmidt MR (2011). Remote preconditioning improves maximal performance in highly trained athletes. Med. Sci. Sports Exerc.

[b13] Jendzjowsky NG, Delorey DS (2013). Short-term exercise training enhances functional sympatholysis through a nitric oxide-dependent mechanism. J. Physiol.

[b14] Jones H, Hopkins N, Bailey TG, Green DJ, Cable NT, Thijssen DH (2014). Seven-Day Remote Ischemic Preconditioning Improves Local and Systemic Endothelial Function and Microcirculation in Healthy Humans. Am. J. Hypertens.

[b15] Keller DM, Ogoh S, Greene S, Olivencia-Yurvati A, Raven PB (2004). Inhibition of KATP channel activity augments baroreflex-mediated vasoconstriction in exercising human skeletal muscle. J. Physiol.

[b16] Kharbanda RK, Peters M, Walton B, Kattenhorn M, Mullen M, Klein N (2001). Ischemic preconditioning prevents endothelial injury and systemic neutrophil activation during ischemia-reperfusion in humans in vivo. Circulation.

[b17] Kimura M, Ueda K, Goto C, Jitsuiki D, Nishioka K, Umemura T (2007). Repetition of ischemic preconditioning augments endothelium-dependent vasodilation in humans: role of endothelium-derived nitric oxide and endothelial progenitor cells. Arterioscler. Thromb. Vasc. Biol.

[b18] Loukogeorgakis SP, Panagiotidou AT, Broadhead MW, Donald A, Deanfield JE, Macallister RJ (2005). Remote ischemic preconditioning provides early and late protection against endothelial ischemia-reperfusion injury in humans: role of the autonomic nervous system. J. Am. Coll. Cardiol.

[b19] Loukogeorgakis SP, Williams R, Panagiotidou AT, Kolvekar SK, Donald A, Cole TJ (2007). Transient limb ischemia induces remote preconditioning and remote postconditioning in humans by a K(ATP)-channel dependent mechanism. Circulation.

[b20] Mancini DM, Bolinger L, Li H, Kendrick K, Chance B, Wilson JR (1994). Validation of near-infrared spectroscopy in humans. J. Appl. Physiol. (1985).

[b21] Miura T, Kawamura S, Tatsuno H, Ikeda Y, Mikami S, Iwamoto H (2001). Ischemic preconditioning attenuates cardiac sympathetic nerve injury via ATP-sensitive potassium channels during myocardial ischemia. Circulation.

[b22] Miyazawa T, Horiuchi M, Ichikawa D, Sato K, Tanaka N, Bailey DM (2012). Kinetics of exercise-induced neural activation; interpretive dilemma of altered cerebral perfusion. Exp. Physiol.

[b23] Mizuno M, Iwamoto GA, Vongpatanasin W, Mitchell JH, Smith SA (2014). Exercise training improves functional sympatholysis in spontaneously hypertensive rats through a nitric oxide-dependent mechanism. Am. J. Physiol. Heart Circ. Physiol.

[b24] Mortensen SP, Nyberg M, Gliemann L, Thaning P, Saltin B, Hellsten Y (2014). Exercise training modulates functional sympatholysis and alpha-adrenergic vasoconstrictor responsiveness in hypertensive and normotensive individuals. J. Physiol.

[b25] Murphy ME, Brayden JE (1995). Nitric oxide hyperpolarizes rabbit mesenteric arteries via ATP-sensitive potassium channels. J. Physiol.

[b26] Murry CE, Jennings RB, Reimer KA (1986). Preconditioning with ischemia: a delay of lethal cell injury in ischemic myocardium. Circulation.

[b27] Parker BA, Smithmyer SL, Jarvis SS, Ridout SJ, Pawelczyk JA, Proctor DN (2007). Evidence for reduced sympatholysis in leg resistance vasculature of healthy older women. Am. J. Physiol. Heart Circ. Physiol.

[b28] Patterson MS, Chance B, Wilson BC (1989). Time resolved reflectance and transmittance for the non-invasive measurement of tissue optical properties. Appl. Opt.

[b29] Pell TJ, Baxter GF, Yellon DM, Drew GM (1998). Renal ischemia preconditions myocardium: role of adenosine receptors and ATP-sensitive potassium channels. Am. J. Physiol.

[b30] Remensnyder JP, Mitchell JH, Sarnoff SJ (1962). Functional sympatholysis during muscular activity. Observations on influence of carotid sinus on oxygen uptake. Circ. Res.

[b31] Shimizu M, Konstantinov IE, Kharbanda RK, Cheung MH, Redington AN (2007). Effects of intermittent lower limb ischaemia on coronary blood flow and coronary resistance in pigs. Acta Physiol. (Oxf).

[b32] Thomas GD, Victor RG (1998). Nitric oxide mediates contraction-induced attenuation of sympathetic vasoconstriction in rat skeletal muscle. J. Physiol.

[b33] Thomas GD, Hansen J, Victor RG (1997). ATP-sensitive potassium channels mediate contraction-induced attenuation of sympathetic vasoconstriction in rat skeletal muscle. J. Clin. Invest.

[b34] Victor RG, Leimbach WN, Seals DR, Wallin BG, Mark AL (1987). Effects of the cold pressor test on muscle sympathetic nerve activity in humans. Hypertension.

[b35] Wray DW, Donato AJ, Nishiyama SK, Richardson RS (2007). Acute sympathetic vasoconstriction at rest and during dynamic exercise in cyclists and sedentary humans. J. Appl. Physiol. (1985).

[b36] Zhou K, Yang B, Zhou XM, Tan CM, Zhao Y, Huang C (2007). Effects of remote ischemic preconditioning on the flow pattern of the left anterior descending coronary artery in normal subjects. Int. J. Cardiol.

